# SLE initially presenting with neuropsychiatric manifestations and seizure, case report

**DOI:** 10.1002/iid3.918

**Published:** 2023-06-28

**Authors:** Abdulrahman Ali M. Khormi, Faisal Taher Hijazi

**Affiliations:** ^1^ Internal Medicine Prince Sattam University Medical College Al‐Kharj‐KSA Al‐Kharj Saudi Arabia; ^2^ Faculty of Medicine Prince Sattam Bin Abdulaziz University Al‐Kharj Saudi Arabia

**Keywords:** autoimmunity, blood, diseases, systemic lupus erythematosus, tissues

## Abstract

Systemic lupus erythematosus (SLE) is a multisystem autoimmune disorder exhibiting variable disease courses and multiple clinical manifestations. SLE's aetiology remains unclear; however, different environmental (e.g., ultraviolet light, infections, drugs, etc.), genetic, and hormonal factors are potentially involved. A positive family history and history of having other autoimmune illnesses are considered high‐risk factors for SLE; nevertheless, most SLE cases are scattered. The 2019 European League Against Rheumatism/American College of Rheumatology classification criteria for SLE include at least one positive antinuclear antibody test as a mandatory entry criterion, followed by additive weighted standards grouped in seven clinical (constitutional, haematological, neuropsychiatric, serosal, musculoskeletal, renal, and mucocutaneous) and three immunological (antiphospholipid antibodies, complement proteins, and SLE‐specific antibodies) domains weighted from 2 to 10, with patients accumulating ≥10 points being diagnosed with SLE. Herein, we report a case of neuropsychiatric lupus, a rare and severe form of SLE.

## Graphical abstract

Main steps at the management of a 16‐year‐old girl with history of neuropsychiatric lupus.

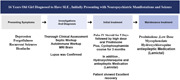

## 1 INTRODUCTION

Systemic lupus erythematosus (SLE) is a multisystem autoimmune disorder exhibiting variable disease courses and multiple clinical manifestations.^1^ SLE's aetiology remains unclear; however, different environmental (e.g., ultraviolet light, infections, drugs, etc.), genetic, and hormonal factors are potentially involved.^1^ A positive family history and history of having other autoimmune illnesses are considered high‐risk factors for SLE; nevertheless, most SLE cases are scattered.^1^ The 2019 European League Against Rheumatism/American College of Rheumatology classification criteria for SLE include at least one positive antinuclear antibody (ANA) test as a mandatory entry criterion, followed by additive weighted standards grouped in seven clinical (constitutional, haematological, neuropsychiatric, serosal, musculoskeletal, renal, and mucocutaneous) and three immunological (antiphospholipid antibodies, complement proteins, and SLE‐specific antibodies) domains weighted from 2 to 10, with patients accumulating ≥10 points being diagnosed with SLE.^2^ Herein, we report a case of neuropsychiatric lupus, a rare and severe form of SLE.

## 2 CASE PRESENTATION

**Figure 1:**
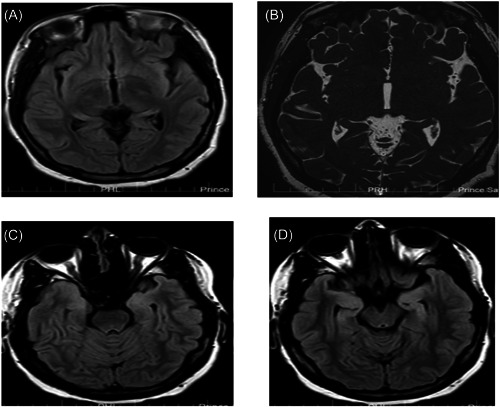
(A–D) MRI brain sections exhibiting prominent sulci, mainly of the Sylvian fissure, corpus callosum atrophy, and bilaterally prominent temporal horns with relatively symmetrical increased FLAIR signal intensities of both hippocampal formations. FLAIR, fluid‐attenuated inversion recovery; MRI, magnetic resonance imaging.

A 16‐year‐old female patient was referred to our rheumatology department with a history of progressive forgetfulness, episodes of jerky movements that were associated with loss of consciousness, upward rotation of both eyeballs, tongue biting, and frothy secretions from the mouth that persisted for 3–10 min, followed by incoherent speech. The patient had complained of a headache over the preceding 12 months. They had no history of cough, vomiting, blurred vision, head injury, anorexia, weight loss, or travel history to a malaria area. The patient neither smoked nor consumed alcohol and was allergic to penicillin. The patient's family history of autoimmune diseases and encephalitis was unremarkable. On receiving treatment in the emergency room of another hospital, the patient was considered a case of suspected viral encephalitis associated with fever; lumbar puncture was performed, an antiviral was administered for 14 days, and the patient was subsequently discharged. Thereafter, the patient continued exhibiting behavioral changes such as depression, increased forgetfulness, and recurrent seizure attacks with agitation for 1 month. At a later date, the patient presented to our clinic and was diagnosed with SLE based on her clinical presentation, which included neurological manifestations and malar rash; moreover, laboratory results revealed ANA and anti‐double‐stranded DNA (dsDNA) antibody positivity. **On initial examination**, the patient appeared agitated yet oriented and had the following vital signs and measurements: pulse rate: 87 beats/min, blood pressure: 166/91 mmHg, respiratory rate: 17/min, temperature: 36°C, and weight: 102 kg. Furthermore, the patient exhibited a photosensitive malar rash, alopecia, lymphadenopathy, and thyroid nodules. Other neurological examination findings were normal, and examination of other systems also revealed no other abnormalities. **Investigations yielded the following**: a complete blood count decrease in white blood cells [2700/µL (4.1–11.6)], an increase in monocytes [14.4% (3.8–11.2)], a normal red blood cell count, and a normal haemoglobin level. Liver function testing revealed a mild elevation of the alanine aminotransferase level [44 (0–40)] and a normal albumin level, while other findings were normal. Kidney function testing yielded creatinine and urea levels of 33 µmol/L (44–80) and 1.95 mmol/L (2.1–7.1), respectively, while other findings were normal. Urine analysis revealed negative blood, protein, and glucose results, and other findings were normal. The erythrocyte sedimentation rate was 7 mm/h (0–20), C‐reactive protein was positive, and rheumatoid factor and anti‐cyclic citrullinated peptide were negative. Antiphospholipid, anti‐SM, and anti‐RNP antibodies were negative. However, anti‐dsDNA antibodies were positive [81.7 IU/mL (positive >35)]. C3 and C4 complement levels were 90 mg/dL (90–180) and 12 mg/dL (12–40), respectively. Thyroid function testing revealed a mild decrease in free thyroid hormone 3 as well as normal thyroid‐stimulating hormone and free thyroid hormone 4 levels. Electrocardiography showed sinus tachycardia. Brain magnetic resonance imaging (MRI) revealed prominent sulci, mainly of the Sylvian fissure; corpus callosum atrophy; and bilaterally prominent temporal horns with relatively symmetrical increased fluid‐attenuated inversion recovery signal intensities of both hippocampal formations (Figure 1A–D). Thyroid ultrasound displayed multiple bilateral nodules with tiny calcific foci. Echocardiography revealed trace aortic and mitral regurgitation, impaired relaxation by tissue Doppler, no pericardial effusion, and normal ventricular dimension. Computed tomography of the chest, abdomen, and pelvis was performed, and the results were unremarkable.

### 2.1 Management

The patient commenced SLE treatment on December 30, 2020, 2 months after initial symptom onset. The patient was initially administered pulse steroid (1000 mg of methylprednisolone) daily for 5 days, followed by oral prednisone 60 mg daily. Subsequently, the patient was administered Plaquenil 400 mg PO OD and cyclophosphamide 2 mg/kg PO OD as well as phenytoin as an antiepileptic medication. However, the patient developed an allergic reaction to phenytoin and was consequently shifted to levetiracetam, became increasingly agitated, and was gradually shifted to Lamictal 25 mg PO BID while being weaned off levetiracetam, thus resolving the agitation. The patient was prescribed Bisoprolol 5 mg PO BID for sinus tachycardia. After 3 months, the patient's medical situation improved, with no more seizures or psychosis. On June 8, 2021, cyclophosphamide was discontinued, CellCept 1000 mg PO BID was commenced, and the steroid dose was gradually reduced to 15 mg PO OD. Since the patient was on steroids, her weight increased, and her lipid profile revealed an increase in total cholesterol and low‐density lipoprotein levels; therefore, she was prescribed atorvastatin and provided with dietary advice. The patient's thyroid‐antibody test result was negative; hence, fine needle aspiration of the thyroid was recommended to check for malignancy, and the biopsy results were unremarkable for malignancy. By the 2nd of March 2022, the patient was on prednisolone 5 mg PO OD, CellCept 1000 mg PO BID, Plaquenil 400 mg PO OD, atorvastatin 10 mg PO OD, Lamictal 25 mg PO BID, Bisoprolol 5 mg PO PD, and Ca and vitamin D tablets. The patient presented no active complaints and appeared to fare well.

### 2.2 Follow‐up

Based on clinical, laboratory, and radiological findings, the diagnosis is SLE manifested primarily with neuropsychiatric symptoms. Lumbar puncture, which had previously been performed, revealed normal cyto‐biochemical markers. Cerebrospinal fluid bacteriological, mycological, and mycobacteriological cultures that had previously been conducted came out negative. On March 7, 2022, the patient was followed up with MRI and electroencephalography (EEG) as well as anti‐dsDNA and C3 and C4 complement assessments. MRI revealed similar changes to those that had initially been observed, with mild signal reductions. Follow up EEG which was done later after recovery revealed no abnormal brain activity. The anti‐dsDNA antibody level decreased to 1.9 IU/mL compared with its initial value of 81.7 IU/mL. The C3 and C4 levels increased to 135 and 26 mg/dL compared with their initial values of 90 and 12 mg/dL, respectively. The patient has been undergoing regular follow‐up with neurology and rheumatology teams. The plan is to taper down the patient's steroid dose to zero. The neurology team will monitor the patient to adjust her antiseizure medication. The patient developed morbid obesity (body mass index >40) and became a candidate for obesity management; therefore, the obesity team will monitor her. Moreover, the patient will also be closely monitored by the rheumatology team for any disease reactivation and medication adjustment.

## 3 DISCUSSION

This case's diagnosis was initially difficult to establish because the main symptoms of central nervous system (CNS) lupus can be diffuse (generalized seizures, psychosis) or focal (stroke, peripheral neuropathies).^3^ Furthermore, the patient's symptoms were vague and nonspecific. Differential diagnosis of our patient's symptoms included hypertensive encephalopathy, toxic leukoencephalopathy caused by therapeutic agents, and metabolic causes involving the nervous system, such as hydro‐electrolytic changes.^3^ The presence of ANAs and anti‐dsDNA antibodies along with the patient's symptoms was a convincing guide toward the final diagnosis. Upon literature review, we identified multiple similar cases. For example, in a case reported by Ferraria et al.,^4^ a 7‐year‐old girl was admitted owing to ataxia, diplopia, and morning vomiting. Brain MRI revealed marked brain lesions; therefore, the patient commenced pulse immunosuppressive treatment followed by psychotropic medications, and azathioprine was initiated as maintenance therapy. Thereafter, the patient exhibited clinical improvement in terms of symptoms and radiology. Another case reported by Iftikhar et al.^5^ involved a 43‐year‐old woman who presented to the emergency department with a seizure. The patient was refractory to conventional antiepileptic medications and later developed a malar rash, at which point SLE diagnosis was established based on refractory positive ANA, arthritis, malar rash, and seizures. Therefore, the patient was administered intravenous methylprednisolone and subsequently maintained by rituximab and oral prednisolone 45 mg, which were tapered gradually. Later, the patient exhibited favorable symptom improvement. In another case reported by Faruk et al.,^6^ a 7‐year‐old girl was admitted to the emergency department due to a seizure. After confirming SLE diagnosis, the patient received intravenous pulse methylprednisolone followed by oral prednisolone. The patient's posttreatment C3 and C4 levels returned to normal, and her symptoms improved.

## 4 CONCLUSION

Continued efforts to further elucidate the pathologic mechanisms underlying various manifestations of SLE and neuropsychiatric features are ongoing to aid in diagnosing and treating neuropsychiatric SLE. In our patient, the combination of clinical features, radiological findings, and positive immunological titres facilitated diagnosis. The patient's symptoms and response to treatment along with her laboratory findings improved, thus ensuring us that we were on the right track. Further studies are obligatory to help rheumatologists appropriately deal with and manage patients with CNS lupus.

## AUTHOR CONTRIBUTIONS

**Abdulrahman Ali M. Khormi**: conceptualization; formal analysis; investigation; project administration; resources; supervision; visualization; writing—original draft; writing—review & editing. **Faisal Taher Hijazi**: data curation; methodology; resources; writing—review & editing.

## CONFLICT OF INTEREST STATEMENT

The authors declare no conflicts of interest.

## ETHICS STATEMENT

The patient consented to the publishing of this case report.

## Data Availability

The data that support the findings of this study are available on request from the corresponding author. The data are not publicly available due to privacy or ethical restrictions.
